# Editorial: Innovative value-based medicine: lessons from China's and APAC's healthcare evolution, volume II

**DOI:** 10.3389/fpubh.2026.1960777

**Published:** 2026-09-25

**Authors:** Jing Yuan, Xiangxiang Jiang, Gang Lv, Z. Kevin Lu

**Affiliations:** 1 State Key Laboratory of Quality Research in Chinese Medicine, Institute of Chinese Medical Sciences, University of Macau, Taipa, Macao SAR, China; 2 Department of Clinical Pharmacy and Outcomes Sciences, College of Pharmacy, University of South Carolina, Columbia, SC, United States; 3 Department of General Surgery, The First Medical Center of Chinese PLA General Hospital, Beijing, China

**Keywords:** Asia-Pacific, China, health policy, health system reform, value-based care, value-based medicine

Value-based medicine (VBM) has moved from a broad aspiration to a concrete reform agenda: health systems are increasingly asked to pursue measurable outcomes rather than clinical activity. The core literature frames value as a relationship among outcomes, patient experience, and resources (1–3), while the Consolidated Health Economic Evaluation Reporting Standards (CHEERS), standard economic-evaluation methods, and health technology assessment guidance show how evidence can be translated into transparent choices (4–6). These tools, however, must be embedded in local institutions rather than applied as abstract checklists. This transition is especially relevant for China and the Asia-Pacific region, where population aging, chronic disease, expensive technologies, and coverage commitments converge. Volume II illustrates that value is not produced by one instrument. It emerges through payment design, price negotiation, resource allocation, hospital operations, outpatient experience, post-acute care, and public health governance. The Research Topic is therefore best read as an evidence base for policy learning and refinement: reforms can improve access and efficiency, but they can also create new inequities and measurement gaps if implementation is not watched closely. This matters because the same policy may look efficient in aggregate yet operate differently across hospitals, consumers, and patient groups.

Payment reform is the clearest thread (Table 1). Xu X. et al. show that payment based on curative-effect value for traditional Chinese medicine (TCM)-dominant anorectal diseases can reduce cost and length of stay while restoring use of TCM-specific diagnostic and treatment services. Sheng et al. report that Diagnosis-Intervention Packet (DIP) payment for type 2 diabetes mellitus lowered hospitalization costs and length of stay but shifted out-of-pocket burden toward resident basic medical insurance enrollees and TCM hospital users. Wu et al. add a warning from Diagnosis-Related Group (DRG) payment data: high-margin cases were more likely to be low-value hospitalizations, suggesting that bundled payment may simply move waste rather than remove it. Zheng et al. find that clinical pathway-based single disease payment for hysterectomy reduced expenses, shortened stays, increased minimally invasive surgery, and lowered complications. Yang et al. complete the implementation picture by showing that clinicians' attitudes, not knowledge alone, shape diagnosis-based payment practice. These studies favor payment models with equity guardrails, case-mix surveillance, and clinician engagement. They also show why average savings should never be interpreted without distributional and behavioral evidence.

**Table 1:** Summary of included articles on value-based medicine in China and the Asia-Pacific region.

Study	Focus	Study design	Main findings	Value-based medicine implications
Xu X. et al.	Payment for traditional Chinese medicine-dominant diseases	Interrupted time-series analysis of 5,304 traditional medicine-dominant anorectal disease inpatients.	Reduced cost and length of stay while restoring traditional medicine service use following value-based payment reform.	Outcome-linked payment can protect clinical distinctiveness while improving efficiency.
Sheng et al.	Diagnosis-Intervention Packet payment and diabetes inpatient care	Interrupted time-series analysis of 45,900 type 2 diabetes inpatient claims.	Reduced hospitalization costs and length of stay but increased out-of-pocket burden for some vulnerable groups.	Payment reform needs explicit equity monitoring, not only spending targets.
Wu et al.	Diagnosis-related group payment and low-value admissions	Claims analysis of 251,811 payment records using multilevel regression.	Low-value hospitalizations rose and were more common among high-margin admissions.	Bundled payment requires safeguards against profitable low-complexity admissions.
Zheng et al.	Clinical pathway-based payment for hysterectomy	Interrupted time-series analysis of 4,727 hysterectomy cases over a decade.	Lower expenses, shorter stays, more minimally invasive surgery, and fewer complications.	Clinical pathways can align cost control with quality improvement.
Yang et al.	Diagnosis-based payment policy knowledge, attitudes, and practices	Cross-sectional survey of 357 professionals in 30 Yunnan hospitals.	Attitudes were stronger drivers of payment practice than knowledge alone.	Implementation success depends on organizational climate and workforce engagement.
Xie et al.	Provincial insurance pooling	China family panel studies analysis using staggered difference-in-differences and inequality decomposition.	Pooling increased inpatient use and spending but did not remove pro-rich inequality.	Fund integration must be paired with resource redistribution.
Xu H. et al.	National drug price negotiation and firm innovation	A-share pharmaceutical firm panel study using multi-period difference-in-differences.	Negotiation access stimulated research investment through market valuation and subsidies.	Price negotiation can create innovation signals when incentives are credible.
Zhu et al.	National drug price negotiation and biopharmaceutical performance	Quarterly panel data from 96 listed biopharmaceutical firms using propensity score matching with difference-in-differences.	Reimbursement-list inclusion increased research investment and patent applications.	Strategic purchasing can coexist with industry innovation.
Yao et al.	Anticancer medicine access	Survey of anticancer medicine availability, price, and affordability.	Innovative anticancer medicines remained less available and affordable.	Value depends on last-mile access, not price policy alone.
Jiang et al.	Lecanemab cost-effectiveness	Lifetime Markov model comparing lecanemab, aducanumab, and standard care.	Lecanemab had better value than aducanumab but was not cost-effective vs. standard care.	High-cost innovation requires price-value alignment.
Liu et al.	Renal denervation value assessment	Multidimensional assessment framework using literature review, focus groups, Delphi, and weighting.	Prioritized efficacy, safety, suitability, patient value, innovation, management, and cost.	Hospital procurement can use multidimensional value criteria.
Sun et al.	Chinese patent medicines for angina	Network meta-analysis, safety grading, and cost-consequence analysis.	Products differed across efficacy, safety, and cost profiles.	Traditional medicine product decisions benefit from multidimensional value assessment.
Huang et al.	Primary healthcare efficiency	Slacks-Based Measure, Directional Distance Function, and Global Malmquist-Luenberger analysis with random forest forecasting.	Efficiency was generally high but regional imbalance and rising demand persisted.	Primary care capacity is foundational for equitable value-based medicine.
Tong Lyu	Resource allocation efficiency	Provincial panel analysis of spatial dependence and distribution dynamics.	Efficiency showed path dependence and club formation across regions.	Targeted support is needed for regions unlikely to converge automatically.
Chin et al.	Outpatient value-based care	Singapore General Hospital case study using a four-stage value-based care framework.	Structured outcome selection and improvement planning supported outpatient value-based care.	Asia-Pacific systems can extend value-based care beyond inpatient and procedural care.
Wei et al.	Patient-centered outpatient satisfaction	Survey of 2,664 outpatients with factor analysis and predictive validation.	Patient-centered care and service processes were major satisfaction drivers.	Patient experience should be treated as a measurable value-based medicine outcome.
Zuo, Zhou, Do, et al.	Home infusion need and demand	Narrative review of demographic, clinical, and public health demand.	Aging, antimicrobial resistance, and prolonged infusion needs support home infusion therapy and home health services.	Home-based post-acute care may reduce hospital burden and improve value.
Zuo, Zhou, Huynh, et al.	Home infusion quality and safety	Narrative review of implementation requirements for home infusion therapy and home health services.	Accreditation, compounding standards, technology, workforce, and reimbursement are prerequisites.	Safe scale-up requires operational infrastructure, not only clinical demand.
Tang et al.	Air pollution health-economic burden	Integrated assessment of fine particulate matter and ozone burden in China from 2013 to 2023.	Fine particulate matter burden declined while ozone-related mortality increased, with regional disparities.	Value-based medicine should include prevention and environmental health gains.

A second group addresses access to medicines and innovation (Table 1). Xie et al. find that provincial pooling of basic medical insurance increased inpatient use and spending without eliminating pro-rich inequality, so financial integration must be linked to resource redistribution. Xu H. et al. and Zhu et al. examine National Drug Price Negotiation from the firm side. Their findings suggest that negotiation access can stimulate research and development investment and patenting, particularly when market signals, subsidies, firm size, and market power support innovation. This challenges the simple claim that price negotiation necessarily discourages industry investment. Yet patient access remains unfinished. Yao et al. show that innovative anticancer medicines were less available and affordable than essential or generic medicines. Jiang et al. report that lecanemab provides better value than aducanumab but is not cost-effective against standard care at current prices. Liu et al. and Sun et al. further show that multidimensional assessment methods can help compare devices and TCM products when evidence, safety, patient value, and budget impact must be balanced.

Delivery capacity is another value determinant (Table 1). Huang et al. estimate generally high primary healthcare efficiency in China, but regional imbalance and projected outpatient demand growth suggest continued pressure on grassroots services. Lyu reaches a similar conclusion by showing spatial dependence, path dependence, and club formation in resource allocation efficiency; weaker regions may not converge without targeted support. Patient-centered implementation is equally important. Chin et al. describe a Singapore General Hospital framework that operationalizes value-based care for outpatient conditions through scoping, outcome selection, baseline assessment, and improvement planning. Wei et al. develop a patient-centered outpatient satisfaction tool and identify the physician-patient relationship, waiting-order management, and service quality as major drivers. These papers remind us that VBM cannot be implemented only through reimbursement rules. It also requires data systems, workflow redesign, institutional learning, and patient-reported measures that capture what care actually feels like. In hospital-centered health systems, this delivery perspective is essential for avoiding narrow, payer-only definitions of value.

Post-acute and environmental studies extend the boundaries of value (Table 1). Zuo, Zhou, Do, et al. argue in Part 1 that home infusion therapy and home health services could address aging, antimicrobial resistance, long infusion needs, and hospital crowding in China. Zuo, Zhou, Huynh, et al. in Part 2 move from demand to execution, emphasizing accreditation, sterile compounding, technology integration, workforce training, and value-aligned reimbursement. Together, these reviews shift the discussion from hospital episodes to longitudinal care delivered where patients live. Tang et al. broaden the frame further by estimating health-economic burdens from fine particulate matter (PM2.5) and ozone (O3) across Chinese regions from 2013 to 2023. The simultaneous decline in PM2.5-related burden and rise in O3-related mortality shows that value is also created through prevention outside clinical encounters. For VBM, the denominator should include avoidable hospital days, delayed institutionalization, exposure risk, and population-level prevention rather than hospital throughput alone.

Taken together, these studies offer several important implications. First, cost containment should not be conflated with value; savings matter only when quality, equity, and outcomes are preserved. Second, price and payment reforms require transparent assessment methods and post-implementation surveillance. Third, VBM in China and the Asia-Pacific region should remain pluralistic: TCM-dominant services, oncology and dementia medicines, medical devices, outpatient pathways, home-based care, and environmental health require distinct evidence standards and operational tools. Fourth, implementation capacity has value in itself. Policies that appear efficient nationally may fail locally when clinicians are unconvinced, data systems are fragmented, or disadvantaged regions lack resources. These constraints are central, not peripheral, to whether evidence-based reform translates to durable practice. Volume II advances the field by presenting VBM as an iterative cycle of measurement, incentive design, learning, and correction. Its practical agenda is clear: define outcomes that matter to patients and society, pay for appropriate care, assess technologies transparently, protect equity, and revise policies when real-world consequences emerge.
